# Comparative immune profiling of pancreatic ductal adenocarcinoma progression among South African patients

**DOI:** 10.1186/s12885-024-12595-x

**Published:** 2024-07-07

**Authors:** Nnenna Elebo, Ebtesam A. Abdel-Shafy, Jones A. O. Omoshoro-Jones, Zanele Nsingwane, Ahmed A. A. Hussein, Martin Smith, Geoffrey Candy, Stefano Cacciatore, Pascaline Fru, Ekene Emmanuel Nweke

**Affiliations:** 1 Department of Surgery, Faculty of Health Sciences, University of Witwatersrand, Johannesburg, 2193 South Africa; 2 Bioinformatics Unit, International Centre for Genetic Engineering and Biotechnology, Observatory, Cape Town, 7925 South Africa; 3 National Research Centre, Cairo, Egypt; 4 Hepatopancreatobiliary Unit, Department of Surgery, Chris Hani-Baragwanath Academic Hospital, Soweto Johannesburg, South Africa; 5 Theodore Bilharz Research Institute, Giza, Egypt; 6 Department of Life and Consumer Sciences, College of Agriculture and Environmental Sciences, University of South Africa, Florida, Roodepoort, South Africa

**Keywords:** Pancreatic ductal adenocarcinoma, PDAC, Immune cells, CD4, CD8, Immunosuppression

## Abstract

**Background:**

Pancreatic Ductal Adenocarcinoma (PDAC) is an aggressive cancer characterized by an immunosuppressive microenvironment. Patients from specific ethnicities and population groups have poorer prognoses than others. Therefore, a better understanding of the immune landscape in such groups is necessary for disease elucidation, predicting patient outcomes and therapeutic targeting. This study investigated the expression of circulating key immune cell markers in South African PDAC patients of African ancestry.

**Methods:**

Blood samples were obtained from a total of 6 healthy volunteers (HC), 6 Chronic Pancreatitis (CP) and 34 PDAC patients consisting of 22 resectable (RPC), 8 locally advanced (LAPC) and 4 metastatic (MPC). Real-time Quantitative Polymerase Chain reactions (RT-qPCR), Metabolomics, Enzyme-Linked Immunosorbent Assay (ELISA), Reactive Oxygen Species (ROS), and Immunophenotyping assays were conducted. Statistical analysis was conducted in R (v 4.3.2). Additional analysis of single-cell RNA data from 20 patients (16 PDAC and 4 controls) was conducted to interrogate the distribution of T-cell and Natural Killer cell populations.

**Results:**

Granulocyte and neutrophil levels were significantly elevated while lymphocytes decreased with PDAC severity. The total percentages of CD3 T-cell subpopulations (helper and double negative T-cells) decreased when compared to HC. Although both NK (*p* = 0.014) and NKT (*p* < 0.001) cell levels increased as the disease progressed, their subsets: NK CD56^dim^CD16^−^ (*p* = 0.024) and NKTs CD56^+^ (*p* = 0.008) cell levels reduced significantly. Of note is the negative association of NK CD56^dim^CD16^−^ (*p* < 0.001) cell levels with survival time. The gene expression analyses showed no statistically significant correlation when comparing the PDAC groups with the controls. The inflammatory status of PDAC was assessed by ROS levels of serum which were elevated in CP (*p* = 0.025), (RPC (*p* = 0.003) and LAPC (*p* = 0.008)) while no significant change was observed in MPC, compared to the HC group. ROS was shown to be positively correlated with GlycA (*R* = 0.45, *p* = 0.0096). Single-cell analyses showed a significant difference in the ratio of NKT cells per total cell counts in LAPC (*p* < 0.001) and MPC (*p* < 0.001) groups compared with HC, confirming observations in our sample group.

**Conclusion:**

The expression of these immune cell markers observed in this pilot study provides insight into their potential roles in tumour progression in the patient group and suggests their potential utility in the development of immunotherapeutic strategies.

**Supplementary Information:**

The online version contains supplementary material available at 10.1186/s12885-024-12595-x.

## Introduction

Pancreatic Ductal Adenocarcinoma (PDAC) is the most common neoplasm of the pancreas with a very poor prognosis and a 5-year survival rate of < 12% [1]. PDAC has been projected to be the second leading cause of cancer worldwide by 2030 [2]. Treatment strategies primarily include chemotherapy, radiotherapy and surgery which may be applied solely or in combination with one another. Currently, surgery is the best clinical treatment for PDAC, however, only about 15–20% of patients will undergo the procedure [3]. Due to the late presentation, most patients are diagnosed with a locally advanced or metastatic stage of the disease which precludes the chance of surgical resection [4]. The tumour microenvironment (TME) of PDAC is characterised by malignant cells, stromal components, and immune cells which converge in a delicate balance [5, 6]. Immune cells have dual roles in PDAC which contribute to tumour progression and paradoxically offer avenues for therapeutic intervention. For example, neutrophils and lymphocytes have been shown to possess pro-tumourigenic and anti-tumourigeneic characteristics, respectively [7]. Furthermore, effector immune cells such as natural killer (NK) cells, CD4^+^ T-cells, and CD8^+^ T-cells are present and activated in the TME at the early stage of PDAC exerting intricate influences on the tumour behaviour either by fueling its growth or orchestrating its suppression [8]. Immunosuppressive mechanisms of PDAC cells include editing the immune system to become unrecognisable leading to tumour escape, activation, and release of immunosuppressive molecules such as IL-10 and TGFβ which inhibit immune response and promote tumour growth and metastasis [9]. Furthermore, tumour cells downregulate the expression of MHC class I molecules by interfering with the antigen cross-presentation to effector T-cells, further exacerbating cancer [10].

The specificity of CD3 antigen for T-cells and its presence at all the stages of T-cell development makes it an ideal T-cell marker for the detection of CD4^+^ T-helper cells and CD8^+^ T-cytotoxic cells. CD4^+^ T-helper cells and CD8^+^ T-cytotoxic cells form large proportions of the T-cells involved in cell-mediated immunity [11, 12]. Alteration of either the number or the function of CD4^+^ T-cells and CD8^+^ T-cells will affect the immune response [13]. Hence maintaining the balance between CD4^+^ and CD8^+^ T-cells is critical for tumour immunity. Cytotoxic lymphocytes play important roles in innate and adaptive immune system response against tumour by secreting cytokines to facilitate their anti-tumour effect [14]. However, elevated levels of cytotoxic CD8^+^ T-cells dysfunction have been observed in advanced stages of PDAC [15]. NK cells represent about 5–25% of circulating lymphocytes and express CD16, CD56, and CD57 markers in humans [16]. The NK populations are distinguished by the markers CD56^bright^ for superior cytokine production [17], CD56^dim^CD16 for immune modulatory function via interferon-γ (IFN-γ) secretion and CD56^dim^CD16^+^ for enhanced cytotoxic abilities [18].

The nexus between immune response, metabolism and inflammation has been widely interrogated in PDAC and has been implicated in tumour progression and treatment response [5, 19–21]. This systematic inflammatory response could be quantified through different scores such as ratios between different circulating immune cells [22] and correlation between immune cells and reactive oxygen species (ROS). Immune cells induce ROS production through the secretion of tumour necrosis factor α (TNF-α) and ROS have been shown to exert an immunosuppressive effect on NK and T-cells [23]. PDAC accumulates ROS which have dual roles depending on their concentration [24, 25]. They can facilitate cancer progression at mild to moderate levels whilst excessive ROS production promotes the release of cytochrome c into the cytoplasm which mediates programmed cell death [26].

In this study, we demonstrate the immune response at both mRNA and protein levels in different stages of PDAC in a group of patients of African ancestry. Studies have shown that the incidence and mortality of PDAC can vary across ethnicity and geographical locations [27, 28]. Importantly, patients of African descent have also been observed to have the poorest prognosis attributed to both genetic, social and environmental factors. Since immune responses could contribute to cancer disparities ), we sought to profile key immune factors in our patient population and highlight their expression patterns.

## Methods

### Patient recruitment

Ethics clearance for this study was obtained from the Human Research Ethics Committee of the University of the Witwatersrand (Study number: M190681). Participants gave written informed consent and were recruited from the Hepatopancreatobiliary Unit at Chris Hani Baragwanath Academic Hospital, Soweto Johannesburg, South Africa. Sample processing was done at the Department of Surgery, Faculty of Health Science, University of the Witwatersrand. Inclusion criteria included patients aged 18 years old and above, self-reported of being of African ancestry, a confirmed clinically and histologically primary diagnosis of one of the three stages of PDAC notably resectable pancreatic cancer (RPC), locally advanced pancreatic cancer (LAPC) and metastatic pancreatic cancer (MPC) according to the American Joint Committee on Cancer (AJCC 8th edition) [29]. For the control groups, patients with chronic pancreatitis (CP) and healthy volunteers (HC) were recruited. All patients in the control groups also self-reported being of African ancestry. Exclusion criteria included patients with organ failure or undergoing any therapy at the time of the study.

### Sampling and Processing

Fasting blood samples were collected by venepuncture in two separate clear vacutainer tubes (BD Biosciences, Franklin Lakes, NJ, USA) with coagulant EDTA and without anti-coagulant. Plasma was obtained by centrifuging the blood at 1734 g, 4 °C for 10 min. The blood was processed to obtain serum by centrifuging at 1734 g, 4 °C for 10 min after allowing it to clot for 30–60 min at room temperature. Peripheral blood mononuclear cells (PBMCs) were separated using the Ficoll-Paque™ (GE Healthcare, Illinois, United States) separation method [30]. PBMCs were collected at a concentration between 10^5^ and 10^6^ cells/ml and stored in a freezing medium (10% dimethyl sulphoxide, Sigma Aldrich, Missouri, USA and 90% Gibco Bovine Serum, Thermo Fischer, Massachusetts, USA) and aliquoted (200 µl) in single-use vials, which were stored at -80 °C until needed. Samples were only thawed once to preserve integrity. One millilitre of diluted BD FACS Lyse (BD Biosciences, New Jersey, United States) solution was added to 100 µl of the whole blood sample to fix the white blood cells. The whole blood FACS lyse mix was allowed to stand at room temperature for 12–15 min after which it was stored at -80ºC until analysis.

Serum, plasma and PBMCs samples were processed within 2 h from the blood collection. An overview of the analysed samples is shown in Table S1. Serum, plasma, PBMCs, lysed whole blood were used to carry out NMR, ROS, Elisa, RT-qPCR and immunophenotyping, respectively (Figure S1).

### Gene expression analysis of immune-related markers

Total RNA was extracted from PBMCs samples, using the TriReagent^®^ (Sigma Aldrich, Missouri, United States) according to the manufacturer’s instructions. The quality of RNA was measured using a NanoDrop ND-1000 Spectrophotometer (Thermo Fischer Scientific, Massachusetts, United States), and A260/280 ratio > 1.8 was observed across all samples. Complimentary DNA (cDNA) synthesis was performed from 250 ng/µl of total RNA using the Photoscript^®^ II First Strand cDNA Synthesis Kit (cat no. E6560S, New England BioLabs^®^ Inc. Massachusetts, United States), according to the manufacturer’s instructions.

A quantitative Real-time Quantitative Polymerase Chain Reaction (RT-qPCR) was then carried out using the TaqMan^®^ Fast Advanced Master Mix (Thermo Fischer, Massachusetts, United States) per the manufacturers’ instructions. The reference gene Microsomal Ribosomal Protein L19 (*MRPL19*) (Hs00608519_m1) [31] and target genes *CD8A* (Hs00233520_m1), *CD4* (Hs01058407_m1), *CD3* (Hs00609515_m1), *CD16/FCGRB* (Hs00275547_m1), *CD56/NCAM1 (*Hs00169851_m1), and *CD57/B3GAT* were obtained from Thermo Fischer Scientific, Massachusetts, United States. The MIQE guidelines were strictly adhered to [32]. The Quant Studio™ 1 Real-Time System (Thermo Fischer Scientific, Massachusetts, United States) was used to run the RT-qPCR reactions.

### Measurement of plasma levels of CD4 and CD8 cellular markers

An immunoassay ELISA kit (Elabscience Biotechnology Inc Houston, USA) which has been pre-coated with antibodies specific to human CD4 and CD8 was used to quantify the concentration of these immune cell markers in the plasma samples. The ELISAs were performed according to the manufacturer’s protocol. Standards and samples were assayed in duplicates and the optical density was determined at 430 nm. The concentrations of CD4 and CD8 (ng/ml) markers were calculated from the standard curve.

### Reactive oxygen species (ROS) assessment

N, N-diethyl-para-phenylenediamine (DEPPD) sulfate is a compound that reacts with the serum to form a coloured cation radical [33]. The amount of radical cation formed is related to the oxidative status of serum and can be expressed as hydrogen peroxide equivalents [34]. ROS can be assessed by measuring the hydrogen peroxide equivalent which is proportional to the absorbance measured spectrophotometrically.

One hundred and forty microlitres of 0.1 M sodium acetate buffer (pH 4.8) was added to each allocated well of a 96-well plate. Five microlitres of serum samples consisting of PDAC and controls as well as standards of different concentrations of hydrogen peroxide solutions: 50, 25, 6.25, 3.13, 1.56, 0.78 and 0.39 µM were added in duplicates. DEPPD and iron sulfate were dissolved in 0.1 M sodium acetate buffer pH 4.8, respectively. One hundred microlitres of the reagent mixture prepared at a ratio of 1:25 was then added to each well. The solution was incubated at 37 °C for 1 min. Colour development was recorded at 505 nm at 25 °C, every 15 s for 30 repeats using an FL 600 Microplate reader Multiscan Sky Microplate Spectrophotometer (Thermo Fisher Scientific, Massachusetts USA).

### Flow cytometry immunophenotyping

Multicolour flow cytometry immunophenotyping analysis was used to determine immune cell populations and frequency. A 6-colour panel was established to characterize heterogeneous cell populations in the blood samples (Table S2). Fully stained samples and unstained samples were prepared from the thawed cells. Stained samples were prepared by adding antibodies in the dark at previously titrated volume and then incubated for 30 min at room temperature. Antibodies were optimized by titration to optimally stain lymphocyte populations and their subpopulations using CD3 BD Horizon Brilliant™ Ultraviolet (BUV), CD4 Alexa flour (AF-700) and CD8 Brilliant Violet™ 605 (BV-605), CD56 PE Phycoerythrin Cyanine 7 (PECy7), CD57 (BB515) while the granulocyte population was stained with CD16 PECy5.

All antibodies were obtained from BD LSRFortessa™ II flow cytometer BD Biosciences (New Jersey, United States). Instrument controls for voltage optimisation using single stained and unstained cells as well cytometer setup and tracking beads assays were performed with each experiment. Compensation controls using compensation beads (Anti-mouse Ig, _K_/Negative control compensation particles set; BD Biosciences, New Jersey, United States) to exclude spillover were also included in addition to the experimental controls of unstained samples [35]. A total of 100,000 events were recorded on the flow cytometer (BD Biosciences, New Jersey, United States). Cells were gated using forward versus side angle light scatter to identify lymphocytes and granulocytes with side scatter versus the various stained markers to confirm these populations as described in Figure S2A. The granulocytes were used to identify CD16^+^ neutrophils (Figure S2B) and lymphocyte subpopulations which are CD3^+^CD56^−^ T-cells, NKT cells and NK cells (Figure S2C). NKT cells were further gated to NKTs CD57^+^ (Figure S2D) and NKTs CD16 (Figure S2E) subsets. NK cells were gated into NK CD57^+^ (Figure S2F), NKCD56^bright^CD16^−^, NKCD56^dim^CD16^−^ and NKCD56^dim^CD16^+^ (Figure S2G). Furthermore, CD3^+^CD56^−^ T-cells were gated into three subsets namely; CD3^+^CD4^−^CD8^−^ (double negative) T-cells (Figure S2H), CD3^+^CD8^+^ (cytotoxic) T cells (Figure S2I) and CD3^+^CD4^+^ (helper) T cells (Figure S2J). The gating strategy used also showed another subpopulation CD8^+^CD57^+^ (Figure S2K) derived from cytotoxic T-cells.

### Single-cell RNA profiling

Publicly available were downloaded from the National Institute of Health Gene Expression Omnibus database under the accession GSE155698. Data were from the PBMCs of 16 PDAC patients (7 Resectable, 5 locally advanced and 4 metastatic) and 4 controls (3 healthy volunteers and 1 duodenal adenoma). The data were processed as indicated in the original publication [15], using the Seurat pipeline version 5.0 [36]. In brief, data were initially filtered and normalized; then variable genes were identified using the FindVariableFeatures function. Data were scaled and centered using linear regression on the counts and the cell cycle score difference was calculated using the CellCycleScoring function. Principal component analysis was used to reduce the dimensionality to 30 principal components and batch effects were corrected using the Harmony algorithm [37]. Differently from the original publication, KODAMA algorithm [38, 39] was used to highlight the sub-population of immune cells. Cell clusters were identified via the FindNeighbors and FindClusters function using a resolution of 1.2–2 for all samples.

### Data analysis

The 2^-ΔΔCT^ method was used to calculate relative changes in gene expression [40]. Statistical analyses and graphical representations of the data were conducted using R (version 4.3.2) and RStudio (2023.9.0.463) software. Comparisons of numerical variables were conducted using Wilcoxon and Kruskal–Wallis rank-sum tests. Fisher’s exact test was utilized to evaluate differences between categorical variables. Spearman’s rank test was employed to compute the correlation coefficient (rho) between variables. Pearson’s correlation coefficient was applied for the correlation matrix. The Wald test was utilized to calculate the statistical significance (p-value) of differences between Kaplan–Meier survival curves. A significance threshold of *p* < 0.05 was adopted. To address multiple testing, a false discovery rate (FDR) of < 10% was applied. The KODAMA algorithm [41] was employed to identify immunophenotyping patterns for flow cytometry results across all samples.

The immunophenotyping data were analysed using FlowJo LLC version 10.8 (BD, Biosciences, New Jersey, United States) using flow cytometry standard (FCS) files linked to the compensation controls from FACSDiva™ software. Cells were gated as singlets to exclude doublets using forward scatter height (FSC-H) and forward side scatter area (FSC-A) parameters. The gating strategies were optimised to identify distinct cell populations based on scatter parameters such as white blood cells into lymphocytes and granulocytes based on forward side scatter (FSC) versus side scatter as well as fluorochrome intensities conjugated to each antibody used. Subsequently, fluorescence histograms and dot plots were generated to visualize the distribution of marker expression within the defined gates. Statistical metrics, including percentages of the total parent, were computed for specific markers. Kaplan–Meier survival curves were created using the R library “survival.” The Wald test was used to calculate the *p*-values between survival curves.

## Results

Forty patients including 22 RPC, 8 LAPC, 4 MPC, and 6 CP as well as six age-matched healthy controls (HC) were recruited in this study. All the healthy participants confirmed that they were in good health and were not taking any regular medication, to be eligible for the study. The clinical parameters, demographics, and comorbidities of this cohort have been reported in our previous study [42]. While type 2 diabetes mellitus, cholangitis, and hypertension were not significantly associated with the disease severity, obstructive jaundice has a higher incidence in patients with LAPC and MPC.

### Effector Immune Cell profiling in PDAC

**Fig. 1:**
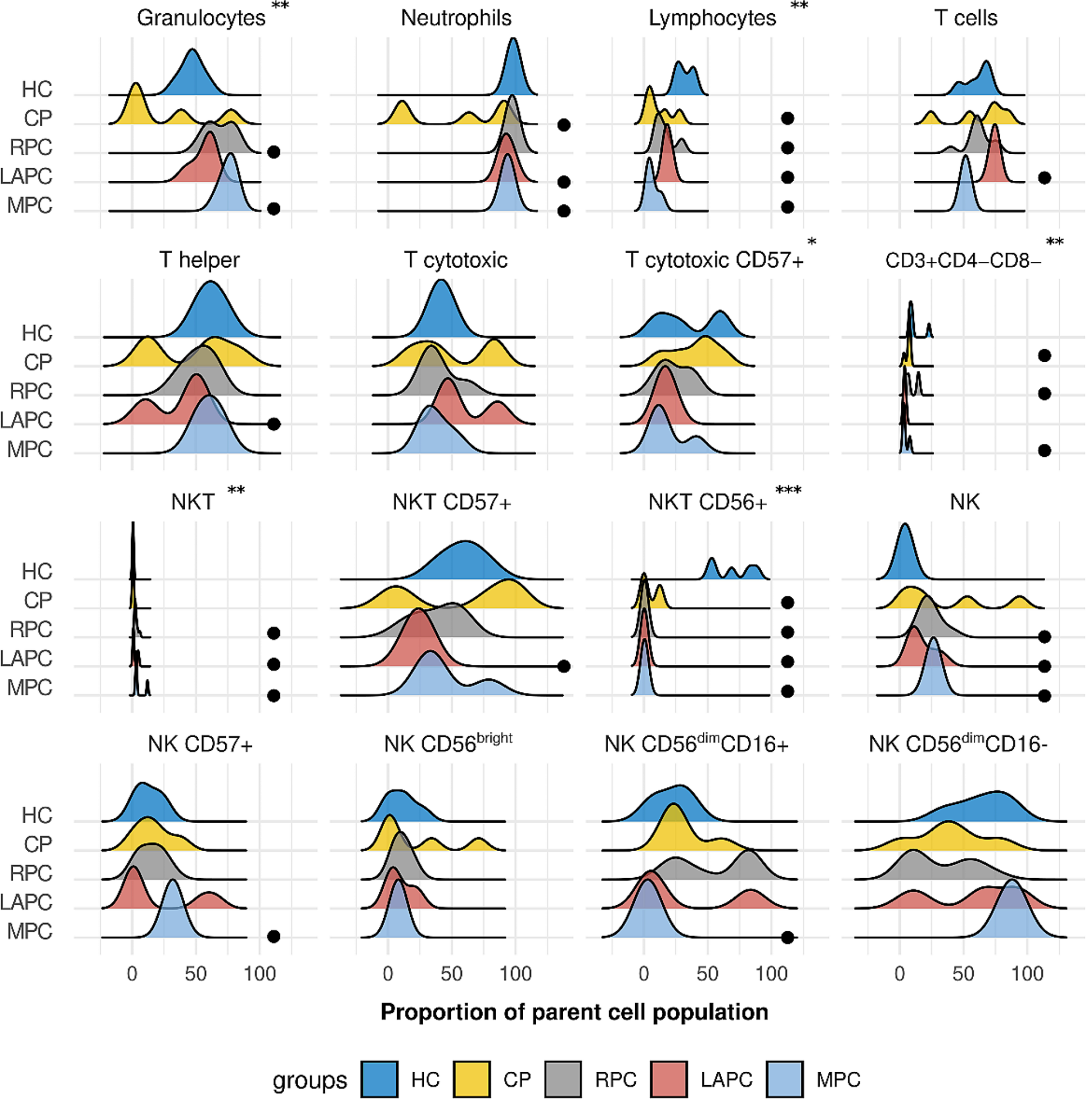
Distribution of effector immune cell populations in PDAC: Granulocytes and neutrophils were distributed to the right indicating elevated levels while lymphocytes shifted to the left signifying decreased levels in PDAC groups. The total parent proportion of CD3 T-cells with increased severity. Subpopulations of CD3 T-cells, T helper and T cytotoxic were also distributed to the left indicating reduction as the disease progressed. However, CD3 subset, cytotoxic T-cell decreased in RPC and MPC but increased in LAPC while it’s subset cytotoxic CD57^+^ T-cell decreased with increased severity and this could be due to it’s enhanced cytotoxicity. There is a shift to the right for NKs and NKTs which signifies elevated levels with increased severity of PDAC. This was not the same for NKT subsets (NKTsCD57^+^ and NKTsCD56^+^) and NK subsets (NKCD56^bright^CD16^−^ NKCD56^dim^CD16^−^ and NKCD56^dim^CD16^+^) which were dysregulated within the PDAC groups. It should be noted that CP control group has an entirely different distribution from both the PDAC and HC groups for all the immune markers. HC; Healthy controls, CP: Chronic Pancreatitis, RPC: Resectable Pancreatic Ductal Adenocarcinoma, LAPC; Locally Advanced Pancreatic Ductal Adenocarcinoma, MPC; Metastatic Pancreatic Ductal Adenocarcinoma *p < 0.05, **p < 0.01, ***p < 0.001. Black circle represent significantly different from HC.

To identify effector immune cell response patterns in PDAC, the immune cell markers CD3, CD4, CD8, CD16, CD56 and CD57 were assessed in the lymphocytes and granulocyte cell populations. CD3, CD4, and CD8 immune cell markers were used to target T-cell lymphocyte subpopulations, CD16 and CD56 were used to determine the NK cell levels, and CD57 for NK cell differentiation [41]. The total percentages of granulocytes significantly increased in PDAC groups compared with HC group ( Table S3). As expected neutrophils were distributed to the right indicating elevated levels because they are the most abundant part of granulocytes, while lymphocytes shifted to the left signifying lower levels in PDAC groups (Fig. 1). Additionally, a shift to the left indicates a decrease in the total percentage of CD3 T-cells with increased severity. Except for CD3^+^ CD8^+^ (cytotoxic T-cell), subpopulations of CD3 T-cells; CD3^+^ CD4^+^ (T helper) and CD3^+^ CD4^−^ CD8^−^ ( Double Negative) T-cells decreased across the PDAC groups when compared to the HCs. Although not significant, HC had the highest percentage of T helper cell subset (Table S3), it should be noted note that the downregulated CD4^+^ T-cell level in this cohort is not related to HIV status because the CD4 counts of these patients were above 300 cells/µl which is within the normal range. These patients are on antiretroviral (ARV) treatments and have normal CD4 counts as shown previously [42]. Cytotoxic T-cell decreased in RPC and MPC but increased in LAPC while its subset cytotoxic CD57^+^ T-cell decreased with increased severity and this could be due to its enhanced cytotoxicity. There is a shift to the right for NKs and NKTs which shows an increase in levels as the disease progresses. Conversely, NKT subsets (NKTsCD57^+^ and NKTsCD56^+^) and NK subsets (NKCD56^bright^CD16^−^, NKCD56^dim^CD16^−^ and NKCD56^dim^CD16^+^) were dysregulated within the PDAC groups. Of importance is the different distribution pattern of CP from both the PDAC and HC groups for all the immune markers.

Additionally, gene and protein expression levels of key markers were assessed using RT-qPCR and ELISA, respectively. Gene expression analysis of the PBMCs showed the differences in fold changes between the PDAC and control groups. There was no significant difference in gene expression profiles across the different groups except for in *CD3* which showed significance when the MPC group was compared to HC (Figure S3). Assessing protein levels, there were significant decreases observed in LAPC and MPC when compared to HC for CD4 marker (Figure S4).

### Characterisation of effector immune cell response in PDAC

**Fig. 2:**
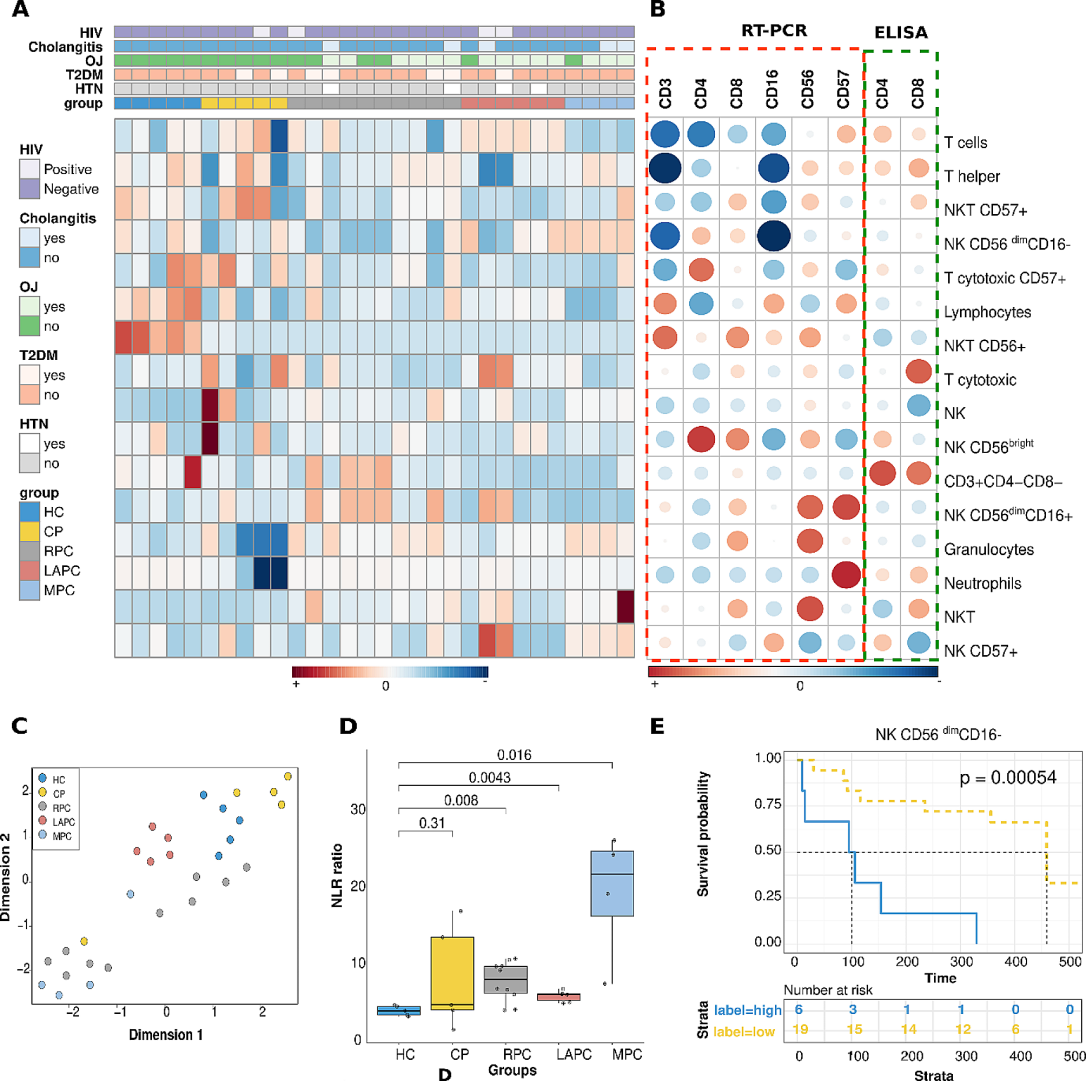
Characterisation of effector immune cell markers in PDAC. **(A)** Heatmap showing the comparison of immune cell profiles across the PDAC groups and their correlation with their comorbidities. NK cells, NK CD56^bright^,NKT cells and NKTs CD56^+^ were observed to have the highest intensities which indicates a strong association with the comorbidities. Granulocytes, neutrophils and T-cells had the weakest intensities which implies no link to the maladies. **(B)** Correlation matrix of Spearman’s rank correlation coefficients between the immune cell populations from Immunophenotyping, RT-qPCR, and Elisa analyses. Dark blue colours indicate strong relationships while dark red signifies weak correlations. **(C)** Unsupervised clustering of the Immunophenotyping data using KODAMA showed that the controls HC and CP were distinctively separated from the PDAC groups. **D)** Comparison of the Neutrophil/Lymphocyte ratio (NLR) between the control and PDAC groups. The NLR was significantly altered across the PDAC groups when compared to the HC group. **(E)** Kaplan Meier plot showing that significant correlation between NK CD56^dim^CD16^−^ cells and patient survival. Patients with lower levels of NK CD56^dim^CD16^−^ cells survived longer. HC; Healthy controls, CP: Chronic Pancreatitis, RPC: Resectable Pancreatic Ductal Adenocarcinoma, LAPC; Locally Advanced Pancreatic Ductal Adenocarcinoma, MPC; Metastatic Pancreatic Ductal Adenocarcinoma

To characterise the immune cell response across the PDAC groups, a heatmap was used to compare their correlation with clinical parameters such as HIV, cholangitis, obstructive jaundice, hypertension and type 2 diabetes (Fig. 2A). NK, NK CD56^bright^, NKT and NKT CD56^+^ cells were observed to have the highest intensities which indicates a strong association with the comorbidities. Granulocytes, neutrophils and T-cells had the weakest intensities which implies no link to the maladies. Furthermore, to further understand the relationship between the immune cells and PDAC progression, the correlation matrix was used to assess the intra-correlation of these immune cells in RT-qPCR, Elisa, and Immunophenotyping analyses (Fig. 2B). The immune cell populations CD3 exhibited a positive correlation with lymphocytes and NKT CD56^+^ cells and lymphocytes. Additionally, neutrophils CD57 were also shown to correlate strongly with both Neutrophils and NK CD56^dim^CD16^+^cells. As expected both CD4 and CD8 were positively linked to T helper, T cytotoxic respectively and CD3^+^CD4^−^CD8^−^ T-cells collectively.

After obtaining the immune matrix, we speculated that these immune cells could distinguish the tumour group from the control group. KODAMA was used to explain the variance-covariance structure of the variable data set through linear combinations of the immunophenotyping data sets to determine the pattern of separation. Except for one CP outlier CP, HC and LAPC, CP, and HC groups were shown to be separate clusters while RPC and MPC were not distinctively separated (Fig. 2C). Prognostic factors for overall survival (OS) were analysed using the Cox proportional hazard regression. Although both CD4/CD8 and neutrophil-lymphocyte ratio (NLR) were not significantly associated with overall survival (OS), the NLR levels increased in RPC, LAPC, and MPC when compared with HC (Fig. 2D). Kaplan Meier survival curve was plotted to correlate the immune cell levels with survival and patients with lower levels of NK CD56^dim^CD16^−^ (*p*-value = 0.00054) cells survived longer (Fig. 2E).

### Immunometabolism and inflammatory response in PDAC

**Fig. 3:**
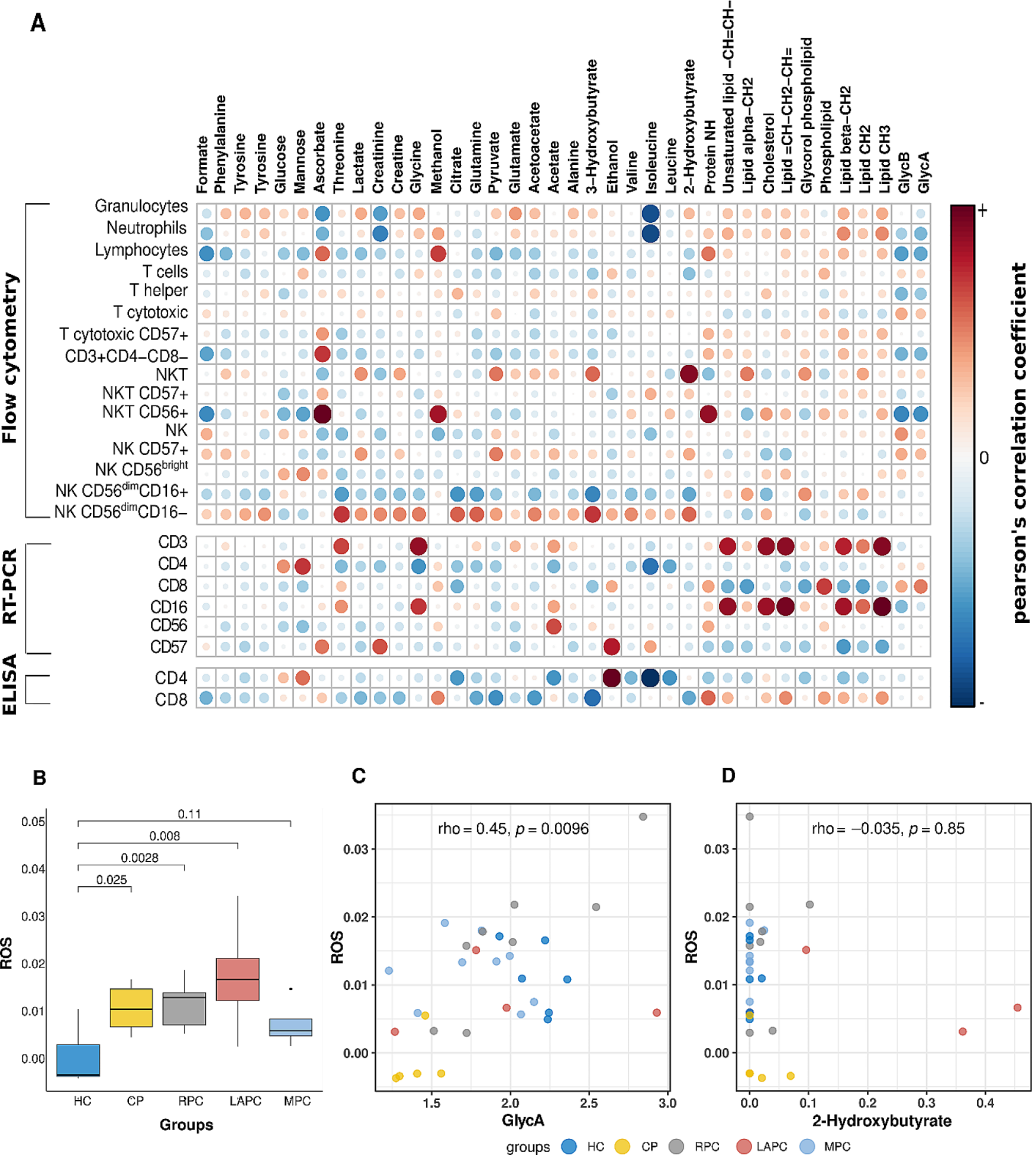
Interplay between immune cell expression, metabolite levels and inflammation in PDAC. **(A)** Correlation matrix between metabolic profile (in columns) and immune profile (in rows) measured by immunophenotyping, RT-qPCR and ELISA. Pearson correlation coefficient was used to measure the linear correlation (red represents positive correlations and blue represents negative correlations **(B)** Boxplot showing the comparison in DEPPD levels representing ROS activity between the PDAC groups (RPC, LAPC, and MPC) and control groups (HC and CP). A significant change was observed when the RPC and LAPC groups of PDAC were compared with HC groups. **(C)** Correlation of ROS and inflammatory marker GlycA. ROS is significantly positively associated with GlycA which is an NMR inflammatory marker. **(D)** Correlation of ROS with 2-hydroxybutyrate. 2-hydroxybutyrate is a metabolite strongly linked to oxidative stress via the impairment of β-cells. Although not significant, there was a negative correlation with ROS. HC; Healthy controls, CP: Chronic Pancreatitis, RPC: Resectable Pancreatic Ductal Adenocarcinoma, LAPC; Locally Advanced Pancreatic Ductal Adenocarcinoma, MPC; Metastatic Pancreatic Ductal Adenocarcinoma

To comprehend the role of metabolism on immune response, quantitative metabolomics analysis was conducted using NMR spectroscopy and several metabolites were observed to be dysregulated in PDAC compared with the control groups as reported in the previous study [42]. Pearson correlation coefficient was used to measure the linear correlation between the two sets; metabolites and immune data obtained from immunophenotyping, Elisa and RT-qPCR. A heatmap was used to interpret the correlation based on the intensities between the immune cells and metabolites (Fig. 3A). Glycine and lipids were observed to be strongly associated with CD3 and CD16 from RT-qPCR assay.

A spectrophotometric assay of ROS showed that there was no significant difference observed when the PDAC groups were compared with CP groups but showed significance when compared with the HC. ROS levels were elevated in RPC (*p*-value = 0.003) and LAPC (*p*-value = 0.008) compared to the HC group (Fig. 3B**)**. Hence, to further understand how and if these metabolites alter the oxidative status of PDAC, ROS levels were correlated with metabolite concentration. Furthermore, a recent study from our laboratory showed that the inflammatory markers GlycA and GlycB were significantly elevated in PDAC patients of African descent when compared to healthy individuals [42]. Hence, GlycA markers (rho = 0.45, *p*-value < 0.0096) were shown to have a significantly positive correlation (Fig. 3C) while 2-hydroxybutyrate, a metabolite linked to oxidative stress (rho = − 0.035, *p*-value = 0.85) although not significant, was inversely associated with the ROS levels (Fig. 3D). To determine if ROS is a good marker of inflammation, a receiver observing characteristic (ROC) curve was plotted with an area under the curve (AUC) value of 0.91 shown in Figure S5.

### T-cell profiling using single-cell RNA sequencing data

**Fig. 4:**
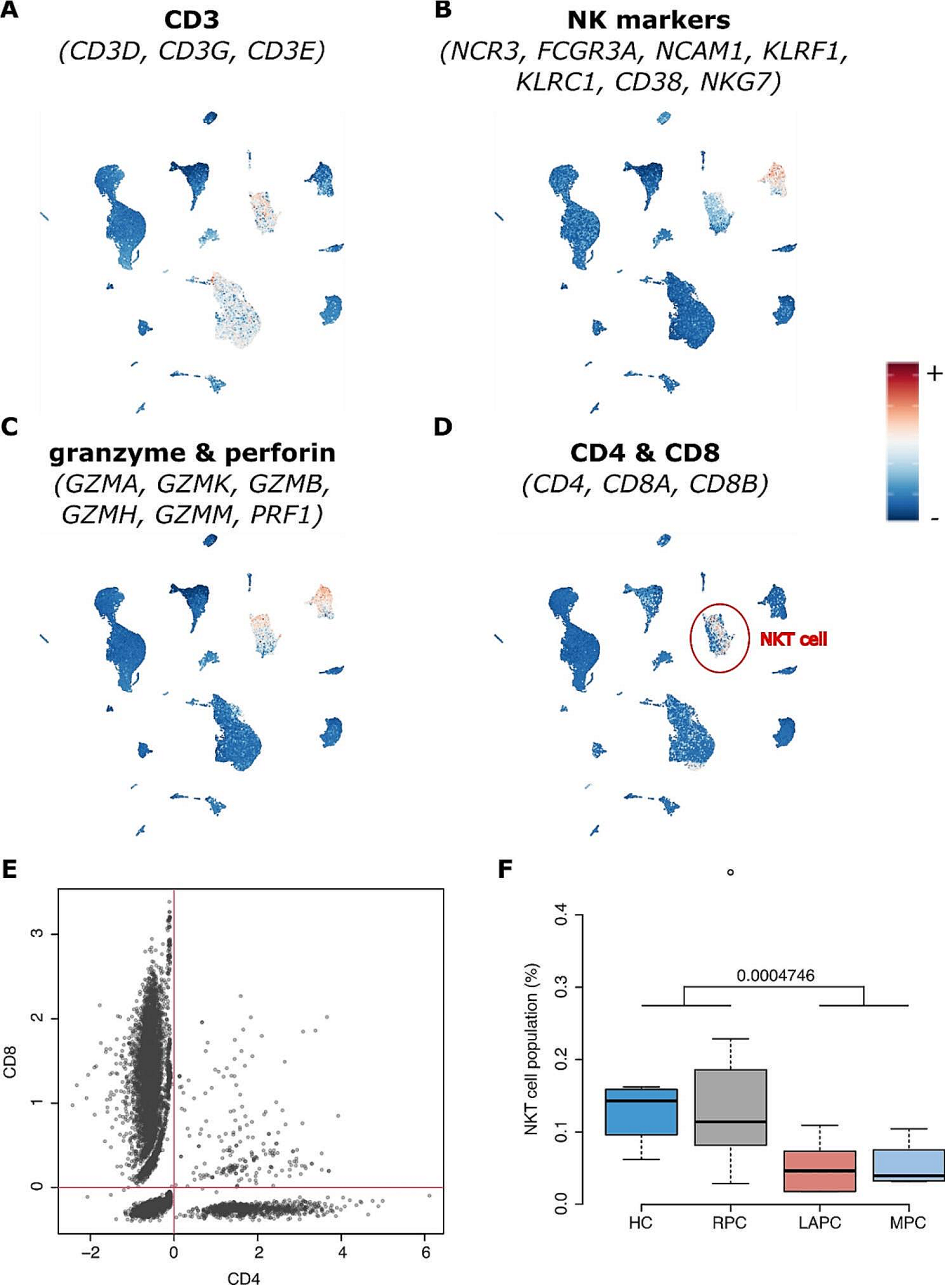
Single-cell analysis of PMBC samples from PDAC patients and HC. Different panels of genes were used to identify **(A)** CD3 expression, **(B)** NK phenotype, **(C)** cytotoxicity activity of granzyme and perforin, **(D)** CD4 + and CD8 + phenotype. **E)** A scatter plot showing the distribution of CD4 and CD8 marker genes in the preidentified NKT cells. **F)** A box plot showing differences NKT cell per total cell counts in PDAC groups (i.e., RPC, LAPC and MPC) and HC. HC; Healthy controls, CP: Chronic Pancreatitis, RPC: Resectable Pancreatic Ductal Adenocarcinoma, LAPC; Locally Advanced Pancreatic Ductal Adenocarcinoma, MPC; Metastatic Pancreatic Ductal Adenocarcinoma

Single-cell RNA sequencing data conducted on PDAC patient PBMCs sourced from Steele et al.‘s (2021) study was utilized to explore T-cell and natural killer (NK) cell populations across different stages of cancer. Unsupervised clustering techniques for PBMC single-cell RNA sequencing (scRNA-seq) data were leveraged via KODAMA, and two dominant clusters primarily of CD3 + T cells were identified (Fig. 4A). One of these clusters exhibited concurrent expression of NK cell gene markers, including NCR3, FCGR3A, NCAM1, KLRF1, KLRC1, CD38, and NKG7 genes (Fig. 4B). Furthermore, cellular cytotoxicity within these clusters was highlighted by monitoring the activity of granzyme and perforin through the expression of GZMA, GZMK, GZMB, GZMH, GZMM, and PRF1 genes (Fig. 4C). The distribution of CD4 and CD8 T cell populations was discerned through the expression analysis of CD4, CD8A, and CD8 genes, aiding in the differentiation between natural killer T (NKT) and NK cells (Fig. 4D). A heterogeneous T-cell population within the pre-identified NKT cells was also identified, showcasing CD4+, CD8+, double-positive, and double-negative NKT cells (Fig. 4E). Finally, a significant decrease in the NKT population previously associated with cytotoxic activity in LAPC and MPC patients was revealed compared to healthy controls and RPC patients (Fig. 4F). These findings shed light on the complex dynamics of T-cell and NK cell populations in PDAC patients across various disease stages.

## Discussion

PDAC tumour cells exhibit immunosuppressive characteristics [43]. Infiltrating immune cells may influence PDAC progression in diverse ways. Hence, the immune status could be essential in predicting the outcome and management of the disease. This study showed that granulocyte levels increased as PDAC progressed from RPC, LAPC to MPC. Recent studies have demonstrated that granulocytes, neutrophils, lymphocytes, and NLR are associated with the overall survival of PDAC patients [7]. However, the granulocyte count is an independent predictive factor for PDAC [7]. Neutrophils contribute to the majority of the granulocyte population; hence it is reasonable to expect that an elevated granulocyte count in this cohort is a consequence of elevated neutrophil levels with PDAC severity. Neutrophils are the main component of chronic inflammation which promotes tumour initiation and progression [44].

CD16^+^ neutrophils (also known as FcγRIIIb) were observed to increase with the disease severity. Neutrophils can exert both pro and anti-tumoural functions which could depend on the type of tumour and microenvironment [44]. Additionally, they exhibit functional plasticity depending on the expression of cell surface markers, cytokines, and ROS. Studies have shown that ROS production is vital in several neutrophil effector functions [45]. Neutrophils contribute to the destruction of cancer cells particularly upon treatment with anti-cancer antibodies, however, the existence of immature neutrophils in circulation mediates immunosuppression and subsequent metastasis [45]. Neutrophil apoptosis is associated with reduced responsiveness and inhibition of receptors activating effector function [46] and loss of its ability to secrete granule enzymes on deliberate external stimulation [47]. CD16^+^ neutrophils uniquely function as an inhibitor of antibody-dependent destruction of cancer cells, thereby identifying it as a potential target for enhancing the therapeutic efficacy of cancer therapeutic antibodies [48]. Furthermore, this study confirms that high NLR suggests a poor prognosis for patients with PDAC and that reduced lymphocyte count is negatively associated with survival rate [7], hence could be used as a novel survival assessment marker [49].

Pancreatic cancer cells downregulate the immune responses by causing a reduction in total lymphocytes and T helper cells which play a crucial role in immune regulation [50]. Although LAPC have elevated levels of cytotoxic T cells compared to HC, lymphocytes decreased with the severity of the disease in this cohort. Additionally, no statistical alteration was recorded between CD4^+^T-cells and tumour severity for HIV-positive PDAC patients in this cohort. Pancreatic cancer cells escape immunity by secreting cytokines such as IL-10 and TGF-β, immunosuppression as a result of these cytokines affects the immune function by inhibiting the infiltration of CD4^+^ and CD8^+^ T cells in the cancer cells [51]. Additionally, DN (CD3^+^ CD4^−^ CD8^−^) T-cells levels, reduced significantly in LAPC and MPC in this cohort. The DN T-cells have been shown to inhibit proliferation and invasion in human pancreatic cancer cells via the Fas/FasL pathway which induces cell apoptosis [52]. Although the detection of Tregs characterized as CD3 + CD4 + CD25 + FOXP3high, was not included in the scope of this study due to resource constraints and the focus on other immune cell populations, they are crucial in maintaining immune homeostasis and contribute to immune evasion within the tumor microenvironment [53, 54].

In this study, NK and NKT cell levels were significantly elevated as the disease progressed. Furthermore, NKT CD16^+^ cells were significantly reduced across the groups. NK cell activation is accompanied by the secretion of inflammatory cytokines thereby driving inflammation which restricts adaptive immune responses [55]. NK cells excluded from PDAC tumours display downregulation of both CD16^+^ and CD57^+^ [56]. NK cells fail to survive or proliferate in a hypoxic microenvironment which contributes to the immune escape of NK cells in PDAC patients.

CD56^+^ drives the maturation of NK cells and is weak in cytotoxicity but strong in the production of anti-tumour cytokines such as IFN-γ and TNF-α [57]. However, although not significant the two main CD56^+^ NK subset populations which are; CD56^bright^ and CD56^dim^ CD16^−^ levels were shown to reduce in LAPC and MPC when compared to HC. This might be due to the inhibited immunomodulatory function and cytotoxic capacity of both subsets respectively [18]. CD57^+^ NK cells are regarded as a marker of terminal differentiation which is less proliferative but more cytotoxic to tumour cells and could acquire IFN-γ when crosslinked with CD16^+^ [58, 59]. Hence might suggest their reduction in PDAC groups compared to the controls. Most of the PDAC patients in this cohort have one or more comorbidities. This study confirmed the strong association between NK cells and Type 2 Diabetes. Dysregulated NK cell responses have been associated with a risk of cardiovascular diseases [60]. Additionally, decreased NK levels are favourable in obstructive jaundice because they lower plasma alanine transaminase and bilirubin levels [61]. Furthermore, DN T-cells were observed to be strongly associated with the comorbidities. Studies have shown that HIV patients undergoing antiretroviral therapy have elevated levels of DN T-cells [62].

Interestingly this study showed that reduced levels of NK CD56^dim^CD16^−^ cells are associated with longer survival time in PDAC. However recent studies show an increased proportion of NK CD56^dim^CD16^−^ in advanced stages of breast cancer [63]. In this cohort, NK CD56^dim^CD16^−^ cell levels decreased in RPC but increased in LAPC and MPC when compared to HC.

Metabolites in tumour microenvironment have been shown to influence immune cell differentiation and effector functions [64]. Tumour cells compete for and deplete essential nutrients that are required for immune cell response [64]. In this study, glycine and lipids were shown to have a strong link with CD3 and CD16, but a poor association with granulocytes and neutrophils. Studies showed that glycine inhibits the calcium flux required for the activation and proliferation of T-lymphocytes [65]. Lipid peroxidation is an important part of lipid metabolism which is vital in signal transduction to control proliferation, differentiation and cell death. The products of lipid peroxidation inhibit T-cell activation via the T-cell receptor (TCR) pathway [66].

ROS can either be detrimental or beneficial for immune cell function and response [67]. In this study, the expression levels of ROS and immune cell markers PDAC progression were evaluated in comparison to control groups consisting HC and CP. In pancreatic cancer, there is an intricate correlation between inflammation and oxidative stress. The chronic inflammatory environment characteristic of pancreatic cancer contributes to the generation of reactive oxygen species (ROS) and reactive nitrogen species (RNS), leading to oxidative stress [68]. We measured ROS as an indicator for oxidative stress, observing significant increases in CP and PDAC groups compared to controls, with significantly lower levels in MPC. This suggests a delicate balance of ROS in PDAC cells, promoting proliferation while preventing senescence and cell death, potentially leading to decreased ROS levels at the MPC stage [69]. Understanding this interplay holds promise for novel therapeutic strategies. ROS promotes apoptosis and cancer cell survival depending on its concentration and cancer cell type [70]. This study confirms that ROS is strongly associated with inflammation because they have closely related pathophysiological activities that are linked [71, 72]. ROS acts as an inflammatory regulator via the activation of NF-_k_B which promotes the expression of proinflammatory cytokines [73].

To delve deeper into the impact of ROS on the inflammatory process, we correlated it with GlycA levels [74]. A positive association between ROS and GlycA was observed, highlighting their involvement in inflammatory mechanisms of tumor progression. Conversely, no significant correlation was found between ROS and 2-hydroxybutyrate (2-HB), a significant metabolite associated with oxidative stress and a marker for cellular redox imbalance and mitochondrial dysfunction [75, 76]. Studies have shown that 2-HB is synthesized in response to oxidative stress and hence a biomarker for screening β-cell dysfunction and hyperglycemia [77] which are all associated with increased risk for PDAC [75]. Further studies are warranted to elucidate different mechanisms contributing to oxidative stress, as indicated by the absence of correlation between ROS and 2-HB.

Through the scrutiny of a single-cell approach for the comprehensive examination of rare cell populations and various immune cell subsets, intricate details about immune response dynamics in PDAC can be uncovered [78, 79]. In our exploration of the immunological landscape within the PBMC scRNA seq dataset, a heterogeneous population of NKT cells was found. Interestingly, remarkable cytotoxic activity was exhibited by these NKT cells. A noteworthy depletion in cytotoxic NKT cells was found in advanced cancer stages either LAPC or MPC, indicating diminished cytotoxic ability with tumor progression. The complexity of the immune response in pancreatic cancer is underscored by this heterogeneity, highlighting the potential importance of these distinct NKT cell subsets in tumor progression.

Sample size was the major limitation in this study which needs to be expanded on in future research. Additionally, the availability of financial resources due to limited funding also played a pivotal role in patient recruitment and some analyses conducted such as immunophenotyping. However, sample collection is ongoing to validate these findings in an increased sample size. The comprehensiveness of immunophenotyping is also limited by the detection ability of flow cytometry. Single-cell RNA sequencing of PBMC samples can further shed light on the key mechanisms underlying immune evasion in pancreatic cancer.

## Conclusion

Effector immune cells could be essential in predicting prognosis in the PDAC cohort. This study showed that increased granulocytes and neutrophil levels, and decreased T-lymphocyte are associated with better outcomes. NK and NKT cell levels correlate with patient survival in this cohort. Evaluating the role of these immune cells as well as their interaction with ROS might be crucial in understanding disease progression and in developing novel therapeutic strategies. The small number of recruited patients in each stage is a limitation. However, to our knowledge, this is the first study of its kind in the study population, providing valuable data in this group of patients. Future studies must incorporate a larger patient cohort and further investigate the interplay between the immune cells in inflammation in the tumour microenvironment.

### Electronic supplementary material

Below is the link to the electronic supplementary material.

Supplementary Material 1

Supplementary Material 2

## Data Availability

The datasets used and/or analysed during the current study are available from the corresponding author on reasonable request.

## Abbreviations

AUC: Area Under The Curve
ARV: Antiretroviral Virus
DEPPD: N, N- Diethyl -Para-Phenylenediamine
DNT: Double Negative T-cells
ELISA: Enzyme-Linked Immunosorbent Assay
FACS: Fluorescence-assisted Cell Sorting
FSC-A: Forward Side Scatter Area
FSH: Forward Scatter Height
GlycA: N-acetylglucosamine
GlycB: Sialic Acid
HC: Healthy Control
HDAC: Histone Deacetylase
IFN-α: Interferon-alpha
Keap1: Kelch-like ECH-associated protein1
PBMC: Peripheral Blood Mononuclear Cells
PDAC: Pancreatic Ductal Adenocarcinoma
Rho: Spearman’s Correlation Coefficient
ROC: Receiver Observing Characteristic
ROS: Reactive Oxygen Species
RT-qPCR: Real-Time Quantitative Polymerase Chain Reaction
LAPC: Locally Advanced Pancreatic Cancer
MHC: Major Histocompatibility Complex
MIQE: Minimum Information For Publication Of Quantitative RT-PCR Experiments
MPC: Metastatic Pancreatic Cancer
MRPL19: Microsomal Ribosomal Protein L19
NF-_k_B: Nuclear Factor Kappa-Light-Chain-Enhancer Of Activated B Cells
Nrf2: Nuclear factor erythroid-derived 2
NK: Natural Killer Cells
NKT: Natural Killer T-cells
NLR: Neutrophil-Lymphocyte Ratio
OS: Overall Survival
RPC: Resectable Pancreatic Cancer
TGFβ: Transforming Growth Factor-beta
TME: Tumor Microenvironment
TNF-α: Tumor Necrosis Factor-alpha

## References

[1] Siegel RL, Miller KD, Wagle NS, Jemal A (2023). Cancer statistics, 2023. Cancer J Clin.

[2] Rahib L, Smith BD, Aizenberg R, Rosenzweig AB, Fleshman JM, Matrisian LM (2014). Projecting Cancer incidence and deaths to 2030: the unexpected burden of thyroid, liver, and pancreas cancers in the United States. Cancer Res.

[3] Wei K, Hackert T. Surgical Treatment of Pancreatic Ductal Adenocarcinoma. Cancers. 2021;13(8). 10.3390/cancers13081971 PMC8074119 33923884

[4] Hingorani SR (2023). Epithelial and stromal co-evolution and complicity in pancreatic cancer. Nat Rev Cancer.

[5] Elebo N, Fru P, Omoshoro-Jones J, Patrick Candy G, Nweke EE (2020). Role of different immune cells and metabolic pathways in modulating the immune response in pancreatic cancer (review). Mol Med Rep.

[6] Nsingwane Z, Candy G, Devar J, Omoshoro-Jones J, Smith M, Nweke E (2020). Immunotherapeutic strategies in pancreatic ductal adenocarcinoma (PDAC): current perspectives and future prospects. Mol Biol Rep.

[7] Feng L, Gu S, Wang P, Chen H, Chen Z, Meng Z et al. White Blood Cell and Granulocyte Counts Are Independent Predictive Factors for Prognosis of Advanced Pancreatic Caner. M’Koma A, editor. Gastroenterology Research and Practice. 2018;2018:8096234. 10.1155/2018/8096234 PMC5964577 29853866

[8] Chang JH, Jiang Y, Pillarisetty VG (2016). Role of immune cells in pancreatic cancer from bench to clinical application: an updated review. Med (Baltim).

[9] Xiang H, Yang R, Tu J, Xi Y, Yang S, Lv L (2023). Metabolic reprogramming of immune cells in pancreatic cancer progression. Biomed Pharmacother.

[10] Garrido F, Perea F, Bernal M, Sánchez-Palencia A, Aptsiauri N, Ruiz-Cabello F. The escape of Cancer from T cell-mediated Immune Surveillance: HLA Class I loss and tumor tissue Architecture. Vaccines. 2017;5(1). 10.3390/vaccines5010007 PMC5371743 28264447

[11] Yang F, Feng C, Zhang X, Lu J, Zhao Y (2017). The Diverse Biological functions of neutrophils, beyond the Defense Against Infections. Inflammation.

[12] Menon AP, Moreno B, Meraviglia-Crivelli D, Nonatelli F, Villanueva H, Barainka M et al. Modulating T cell responses by targeting CD3. Cancers. 2023;15(4). 10.3390/cancers15041189 PMC9953819 36831533

[13] Riazi Rad F, Ajdary S, Omranipour R, Alimohammadian MH, Hassan ZM (2015). Comparative analysis of CD4 + and CD8 + T cells in tumor tissues, lymph nodes and the peripheral blood from patients with breast cancer. Iran Biomed J.

[14] Paul S, Lal G (2017). The molecular mechanism of natural killer cells function and its importance in Cancer Immunotherapy. Front Immunol.

[15] Steele NG, Carpenter ES, Kemp SB, Sirihorachai VR, The S, Delrosario L (2020). Multimodal mapping of the tumor and peripheral blood immune landscape in human pancreatic cancer. Nat Cancer.

[16] Abel AM, Yang C, Thakar MS, Malarkannan S (2018). Natural killer cells: Development, Maturation, and clinical utilization. Front Immunol.

[17] Vujanovic L, Chuckran C, Lin Y, Ding F, Sander CA, Santos PM (2019). CD56^dim^ CD16 Natural Killer Cell Profiling in Melanoma patients receiving a Cancer Vaccine and Interferon-α. Front Immunol.

[18] Granzin M, Wagner J, Köhl U, Cerwenka A, Huppert V, Ullrich E (2017). Shaping of Natural Killer Cell Antitumor activity by *Ex vivo* cultivation. Front Immunol.

[19] Wörmann SM, Diakopoulos KN, Lesina M, Algül H (2013). The immune network in pancreatic cancer development and progression. Oncogene.

[20] Fru PN, Nweke EE, Augustine TN. Harnessing the Tumor Microenvironment for Cancer Immunotherapy. In: Rezaei N, editor. Handbook of Cancer and Immunology [Internet]. Cham: Springer International Publishing; 2022. pp. 1–25. 10.1007/978-3-030-80962-1_183-1.

[21] Zanele Nsingwane P, Naicker J, Omoshoro-Jones J, Devar M, Smith G, Candy (2024). Inhibition of the complement pathway induces Cellular Proliferation and Migration in Pancreatic Ductal Adenocarcinoma. J Biol Regul Homeost Agents.

[22] Schlanger D, Popa C, Pașca S, Seicean A, Al Hajjar N (2022). The role of systemic immuno-inflammatory factors in resectable pancreatic adenocarcinoma: a cohort retrospective study. World J Surg Oncol.

[23] Oberkampf M, Guillerey C, Mouriès J, Rosenbaum P, Fayolle C, Bobard A (2018). Mitochondrial reactive oxygen species regulate the induction of CD8(+) T cells by plasmacytoid dendritic cells. Nat Commun.

[24] Cheung EC, DeNicola GM, Nixon C, Blyth K, Labuschagne CF, Tuveson DA (2020). Dynamic ROS Control by TIGAR regulates the initiation and progression of pancreatic Cancer. Cancer Cell.

[25] Park HJ, Choi YJ, Lee JH, Nam MJ (2017). Naringenin causes ASK1-induced apoptosis via reactive oxygen species in human pancreatic cancer cells. Food Chem Toxicol.

[26] Matilla AJ (2021). Cellular oxidative stress in programmed cell death: focusing on chloroplastic 1O2 and mitochondrial cytochrome-c release. J Plant Res.

[27] Kiely M, Lord B, Ambs S (2022). Immune response and inflammation in cancer health disparities. Trends Cancer.

[28] Samaan JS, Abboud Y, Oh J, Jiang Y, Watson R, Park K (2023). Pancreatic Cancer Incidence trends by Race, Ethnicity, Age and Sex in the United States: a Population-based study, 2000–2018. Cancers (Basel).

[29] Amin MB, Greene FL, Edge SB, Compton CC, Gershenwald JE, Brookland RK (2017). The Eighth Edition AJCC Cancer staging Manual: continuing to build a bridge from a population-based to a more personalized approach to cancer staging. CA: A Cancer. J Clin.

[30] Kleiveland CR et al. Peripheral Blood Mononuclear Cells. In: Verhoeckx K, Cotter P, López-Expósito I, Kleiveland C, Lea T, Mackie A, editors. The Impact of Food Bioactives on Health: in vitro and ex vivo models [Internet]. Cham: Springer International Publishing; 2015. pp. 161–7. 10.1007/978-3-319-16104-4_15. 29787039

[31] Mohelnikova-Duchonova B, Oliverius M, Honsova E, Soucek P (2012). Evaluation of reference genes and normalization strategy for quantitative real-time PCR in human pancreatic carcinoma. Dis Markers.

[32] Bustin SA, Benes V, Garson JA, Hellemans J, Huggett J, Kubista M (2009). The MIQE guidelines: Minimum Information for publication of quantitative real-time PCR experiments. Clin Chem.

[33] Hayashi I, Morishita Y, Imai K, Nakamura M, Nakachi K, Hayashi T (2007). High-throughput spectrophotometric assay of reactive oxygen species in serum. Mutat Research/Genetic Toxicol Environ Mutagen.

[34] Verde V, Fogliano V, Ritieni A, Maiani G, Morisco F, Caporaso N (2002). Use of N, N -dimethyl- p -phenylenediamine to evaluate the oxidative status of human plasma. Free Radic Res.

[35] Nalisa M, Nweke EE, Smith MD, Omoshoro-Jones J, Devar JW, Metzger R (2021). Chemokine receptor 8 expression may be linked to disease severity and elevated interleukin 6 secretion in acute pancreatitis. World J Gastrointest Pathophysiol.

[36] Hao Y, Stuart T, Kowalski MH, Choudhary S, Hoffman P, Hartman A (2024). Dictionary learning for integrative, multimodal and scalable single-cell analysis. Nat Biotechnol.

[37] Korsunsky I, Millard N, Fan J, Slowikowski K, Zhang F, Wei K (2019). Fast, sensitive and accurate integration of single-cell data with Harmony. Nat Methods.

[38] Cacciatore S, Luchinat C, Tenori L (2014). Knowledge discovery by accuracy maximization. Proc Natl Acad Sci U S A.

[39] Cacciatore S, Tenori L, Luchinat C, Bennett PR, MacIntyre DA (2017). KODAMA: an R package for knowledge discovery and data mining. Bioinformatics.

[40] Livak KJ, Schmittgen TD (2001). Analysis of relative gene expression data using real-time quantitative PCR and the 2 – ∆∆CT method. Methods.

[41] Herold NC, Mitra P. Immunophenotyping [Internet]. StatPearls Publishing, Treasure Island (FL); 2022. http://europepmc.org/abstract/MED/32644353. 32644353

[42] Elebo N, Omoshoro-Jones J, Fru PN, Devar J, De Wet C, Vorster BC et al. Serum metabolomic and lipoprotein profiling of pancreatic ductal adenocarcinoma patients of African ancestry. Metabolites. 2021;11(10). 10.3390/metabo11100663 PMC8540259 34677378

[43] Foucher ED, Ghigo C, Chouaib S, Galon J, Iovanna J, Olive D (2018). Pancreatic ductal adenocarcinoma: a strong imbalance of good and bad immunological cops in the Tumor Microenvironment. Front Immunol.

[44] Galdiero MR, Varricchi G, Loffredo S, Mantovani A, Marone G (2018). Roles of neutrophils in cancer growth and progression. J Leukoc Biol.

[45] Mackey JBG, Coffelt SB, Carlin LM (2019). Neutrophil Maturity in Cancer. Front Immunol.

[46] Hart SP, Ross JA, Ross K, Haslett C, Dransfield I (2000). Molecular characterization of the surface of apoptotic neutrophils: implications for functional downregulation and recognition by phagocytes. Cell Death Differ.

[47] Haslett C, Savill JS, Whyte MKB, Stern M, Dransfield I, Meagher LC (1994). Granulocyte apoptosis and the control of inflammation. Philosophical Trans Royal Soc Lond Ser B: Biol Sci.

[48] Treffers LW, van Houdt M, Bruggeman CW, Heineke MH, Zhao XW, van der Heijden J (2019). FcγRIIIb restricts antibody-dependent Destruction of Cancer cells by human neutrophils. Front Immunol.

[49] Yang JJ, Hu ZG, Shi WX, Deng T, He SQ, Yuan SG (2015). Prognostic significance of neutrophil to lymphocyte ratio in pancreatic cancer: a meta-analysis. World J Gastroenterol.

[50] Fogar P, Sperti C, Basso D, Sanzari MC, Greco E, Davoli C et al. Decreased Total Lymphocyte Counts in Pancreatic Cancer: An Index of Adverse Outcome. Pancreas [Internet]. 2006;32(1). https://journals.lww.com/pancreasjournal/fulltext/2006/01000/decreased_total_lymphocyte_counts_in_pancreatic.4.aspx. 10.1097/01.mpa.0000188305.90290.50 16340740

[51] Fukunaga A, Miyamoto M, Cho Y, Murakami S, Kawarada Y, Oshikiri T (2004). CD8 + tumor-infiltrating lymphocytes together with CD4 + tumor-infiltrating lymphocytes and dendritic cells improve the prognosis of patients with pancreatic adenocarcinoma. Pancreas.

[52] YIN LU, PIBO HU, HAIBO ZHOU, ZHIJIAN YANG, YU SUN, HOFFMAN ROBERTM (2019). Double-negative T cells inhibit Proliferation and Invasion of Human Pancreatic Cancer cells in co-culture. Anticancer Res.

[53] Huber M, Brehm CU, Gress TM, Buchholz M, Alashkar Alhamwe B, von Strandmann EP (2020). The Immune Microenvironment in Pancreatic Cancer. Int J Mol Sci.

[54] Goulart MR, Stasinos K, Fincham REA, Delvecchio FR, Kocher HM (2021). T cells in pancreatic cancer stroma. World J Gastroenterol.

[55] Zitti B, Bryceson YT (2018). Natural killer cells in inflammation and autoimmunity. Cytokine Growth Factor Rev.

[56] Marcon F, Zuo J, Pearce H, Nicol S, Margielewska-Davies S, Farhat M (2020). NK cells in pancreatic cancer demonstrate impaired cytotoxicity and a regulatory IL-10 phenotype. null.

[57] Zhang S, Liu W, Hu B, Wang P, Lv X, Chen S (2020). Prognostic significance of Tumor-Infiltrating Natural Killer cells in solid tumors: a systematic review and Meta-analysis. Front Immunol.

[58] Kared H, Martelli S, Tan SW, Simoni Y, Chong ML, Yap SH (2018). Adaptive NKG2C(+)CD57(+) natural killer cell and Tim-3 expression during viral infections. Front Immunol.

[59] Kared H, Martelli S, Ng TP, Pender SLF, Larbi A (2016). CD57 in human natural killer cells and T-lymphocytes. Cancer Immunol Immunother.

[60] Mxinwa V, Dludla PV, Nyambuya TM, Mokgalaboni K, Mazibuko-Mbeje SE, Nkambule BB (2020). Natural killer cell levels in adults living with type 2 diabetes: a systematic review and meta-analysis of clinical studies. BMC Immunol.

[61] Squires JE, Shivakumar P, Mourya R, Bessho K, Walters S, Bezerra JA (2015). Natural killer cells promote long-term hepatobiliary inflammation in a low-dose rotavirus model of experimental biliary atresia. PLoS ONE.

[62] Korbi F, Zamali I, Rekik R, Ben Hmid A, Hidri M, Kammoun Rebai W et al. Double-negative T cells are increased in HIV-infected patients under antiretroviral therapy. Medicine [Internet]. 2022;101(36). https://journals.lww.com/md-journal/fulltext/2022/09090/double_negative_t_cells_are_increased_in.45.aspx. 10.1097/MD.0000000000030182 PMC10980362 36086717

[63] Mamessier E, Pradel LC, Thibult ML, Drevet C, Zouine A, Jacquemier J (2013). Peripheral blood NK cells from breast cancer patients are tumor-induced composite subsets. J Immunol.

[64] Xia L, Oyang L, Lin J, Tan S, Han Y, Wu N (2021). The cancer metabolic reprogramming and immune response. Mol Cancer.

[65] Stachlewitz R, Li X, Smith S, Bunzendahl H, Graves L, Thurman R (2000). Glycine inhibits growth of T lymphocytes by an IL-2-independent mechanism. J Immunol.

[66] Xiao L, Xian M, Zhang C, Guo Q, Yi Q (2023). Lipid peroxidation of immune cells in cancer. Front Immunol.

[67] Kotsafti A, Scarpa M, Castagliuolo I, Scarpa M. Reactive oxygen species and Antitumor Immunity—from Surveillance to Evasion. Cancers. 2020;12(7). 10.3390/cancers12071748 PMC7409327 32630174

[68] Zhang L, Li J, Zong L, Chen X, Chen K, Jiang Z (2016). Reactive oxygen species and targeted therapy for pancreatic Cancer. Abdel Moneim AE. Editor Oxidative Med Cell Longev.

[69] Arfin S, Jha NK, Jha SK, Kesari KK, Ruokolainen J, Roychoudhury S (2021). Oxidative stress in Cancer Cell Metabolism. Antioxid (Basel).

[70] Aggarwal V, Tuli HS, Varol A, Thakral F, Yerer MB, Sak K et al. Role of reactive oxygen species in Cancer Progression: Molecular mechanisms and recent advancements. Biomolecules. 2019;9(11). 10.3390/biom9110735 PMC6920770 31766246

[71] Menzel A, Samouda H, Dohet F, Loap S, Ellulu MS, Bohn T. Common and novel markers for measuring inflammation and oxidative stress Ex vivo in Research and Clinical Practice—which to use regarding Disease. Outcomes? Antioxid. 2021;10(3). 10.3390/antiox10030414 PMC8001241 33803155

[72] Biswas SK (2015). Metabolic reprogramming of Immune cells in Cancer Progression. Immunity.

[73] Forrester SJ, Kikuchi DS, Hernandes MS, Xu Q, Griendling KK (2018). Reactive oxygen species in metabolic and inflammatory signaling. Circ Res.

[74] Janakiram NB, Mohammed A, Bryant T, Ritchie R, Stratton N, Jackson L (2017). Loss of natural killer T cells promotes pancreatic cancer in LSL-KrasG12D/+ mice. Immunology.

[75] Wolpin BM, Bao Y, Qian ZR, Wu C, Kraft P, Ogino S (2013). Hyperglycemia, insulin resistance, impaired pancreatic β-Cell function, and risk of pancreatic Cancer. JNCI: J Natl Cancer Inst.

[76] Chriett S, Dąbek A, Wojtala M, Vidal H, Balcerczyk A, Pirola L (2019). Prominent action of butyrate over β-hydroxybutyrate as histone deacetylase inhibitor, transcriptional modulator and anti-inflammatory molecule. Sci Rep.

[77] Gall WE, Beebe K, Lawton KA, Adam KP, Mitchell MW, Nakhle PJ (2010). α-Hydroxybutyrate is an early biomarker of insulin resistance and glucose intolerance in a nondiabetic Population. PLoS ONE.

[78] Werba G, Weissinger D, Kawaler EA, Zhao E, Kalfakakou D, Dhara S (2023). Single-cell RNA sequencing reveals the effects of chemotherapy on human pancreatic adenocarcinoma and its tumor microenvironment. Nat Commun.

[79] Lin W, Noel P, Borazanci EH, Lee J, Amini A, Han IW (2020). Single-cell transcriptome analysis of tumor and stromal compartments of pancreatic ductal adenocarcinoma primary tumors and metastatic lesions. Genome Med.

