# Supplementary material for: Comparative immune profiling of pancreatic ductal adenocarcinoma progression among South African patients
Source: BMC Cancer. 2024 Jul 7;24:809. doi: 10.1186/s12885-024-12595-x (PMC11229237; doi:10.1186/s12885-024-12595-x)
Supplement: Supplementary file 1 — Supplementary Material 1 [file 12885_2024_12595_MOESM1_ESM.docx]

SUPPLEMENTARY FIGURES


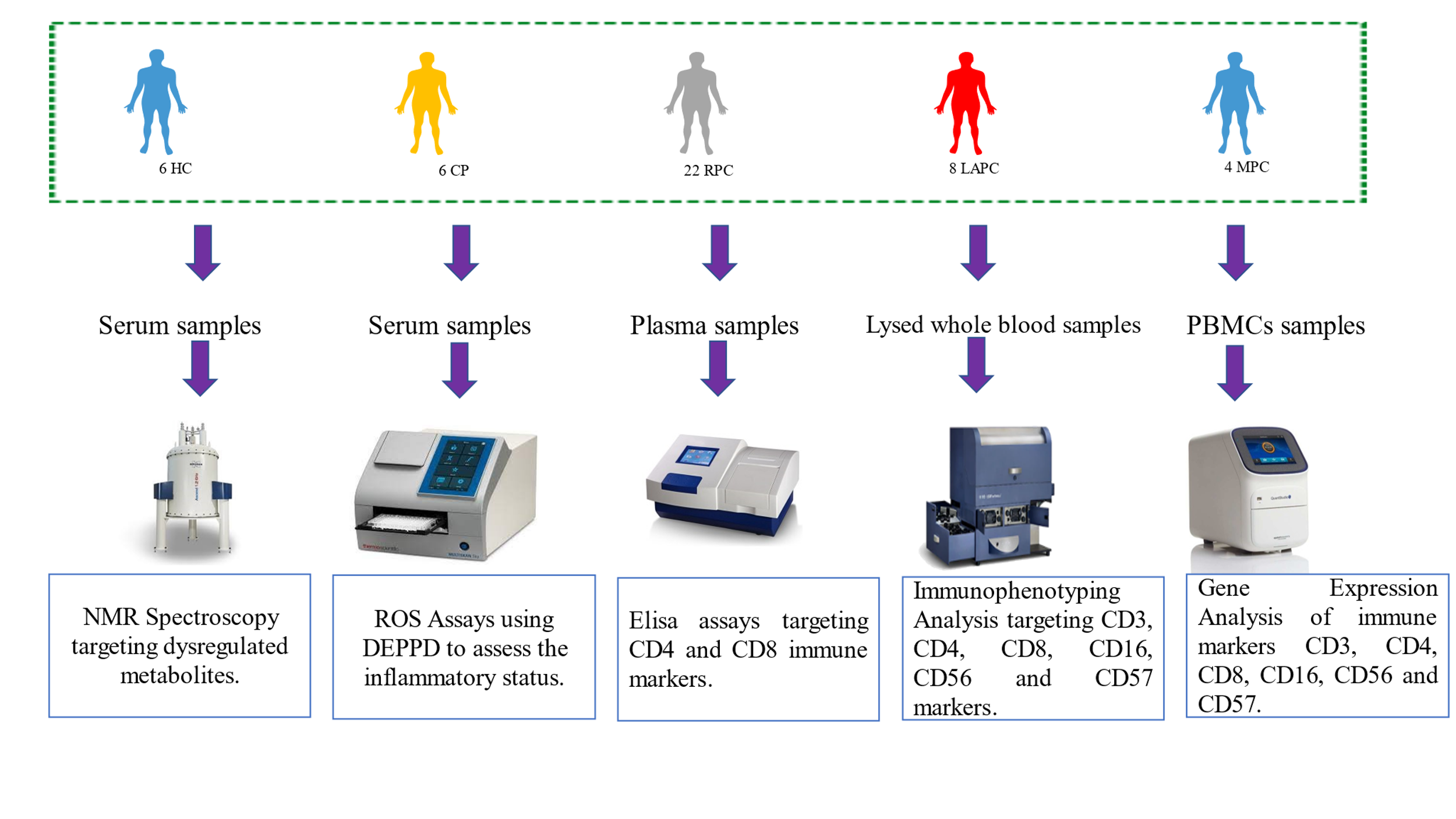


Figure S1: Overview of Sample Types and Assays. After Ethics approval and informed consent blood samples collected from 34 PDAC patients, 6 CP patients, and 6 healthy volunteers were processed into serum for NMR and ROS, plasma for Elisa, lysed whole blood for immunophenotyping and PBMCs for Real-Time Polymerase Chain Reaction Analyses. The same sample was used for all analyses although in varied sample sizes. HC; Healthy controls, CP: Chronic Pancreatitis, RPC: Resectable Pancreatic Ductal Adenocarcinoma, LAPC; Locally Advanced Pancreatic Ductal Adenocarcinoma, MPC; Metastatic Pancreatic Ductal Adenocarcinoma.


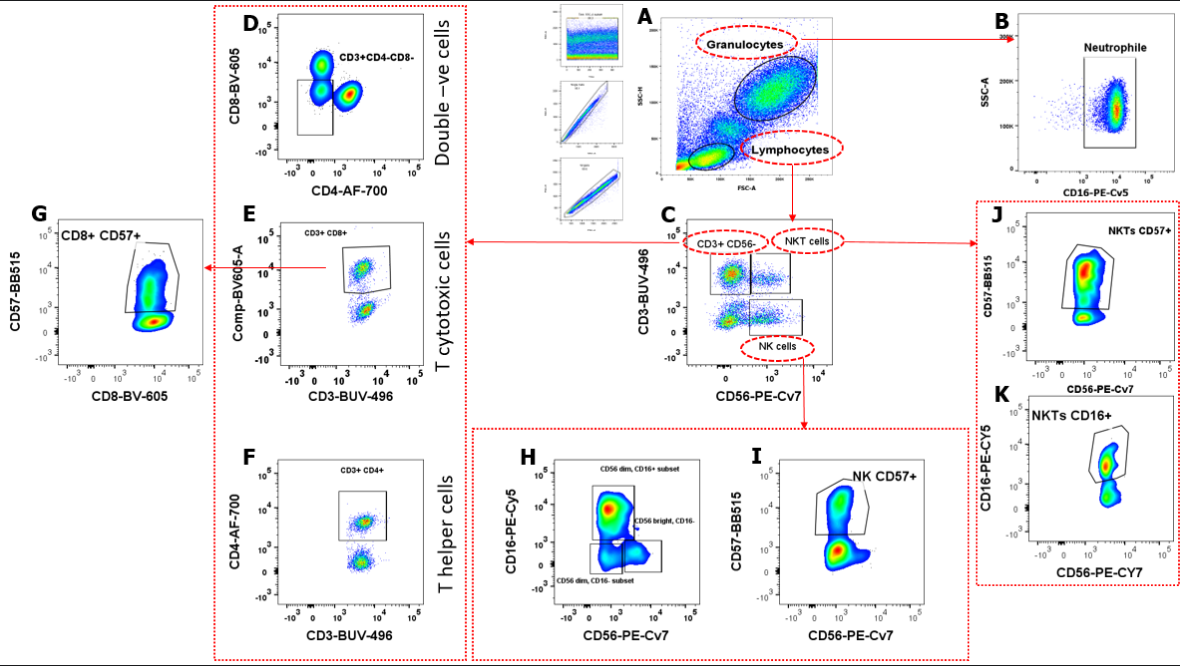


**Figure S2:**  **Gating strategy of the immune cell markers populations and subsets using FlowJo.** **A**. The total number of cell events collected was 100,000. Cells were gated into Singlets (FSC-A versus FSC-H) which were further gated to granulocyte and lymphocyte populations. **B.** Granulocytes are further gated to Neutrophils (SSC-A versus CD16). **C.** Lymphocytes were gated to CD3CD56^+^, NKT-cells and NK cells (CD3 versus CD56) **D and E**. NKT cells were further gated into CD16^+^NKTs subsets (CD57 versus CD56) and CD57^+^NKTs subsets (CD16 versus CD56) respectively. **F and G.** NK cells were further gated to CD57^+^ NK subsets and CD56^+^CD16 NK subsets **H.I.and J.** CD3^+^ T-cells were gated to different subsets; double negative T-cells (CD3^+^CD4^-^CD8^-^), T-cytotoxic cells and T-helper cells. **K**.T-cytotoxic cells were gated into CD8^+^CD57^+^ (CD57 versus CD8). CD3 BD Horizon Brilliant™ Ultraviolet (BUV), CD4 Alexa flour (AF-700) and CD8 Brilliant Violet™ 605 (BV-605), CD56 PE Phycoerythrin Cyanine 7 (PECy7), CD57 (BB515) while the granulocyte population was stained with CD16 PECy5.


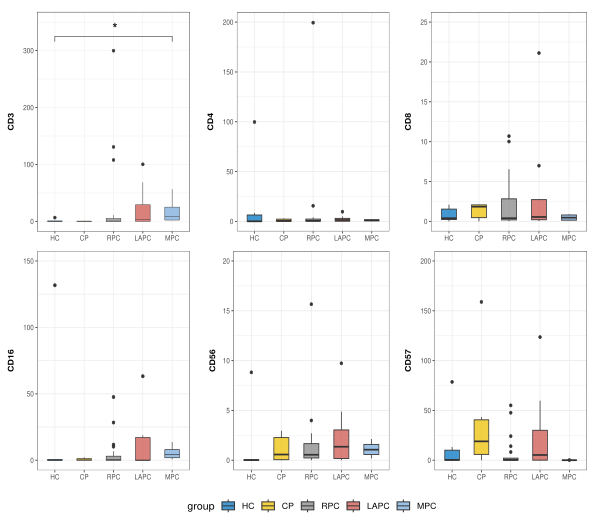


Figure S3: Graphical representation of the immune marker by gene expression. CD8, CD16, and CD56 genes were upregulated in LAPC. HC, RPC, and CP have the highest levels of CD4, CD3, and CD57 genes respectively. Although there was no statistical correlation or differences observed when comparing the PDAC groups with the control groups. CD3, CD4 and CD56 genes were observed to be most upregulated in RPC and CD8 was highest in LAPC.


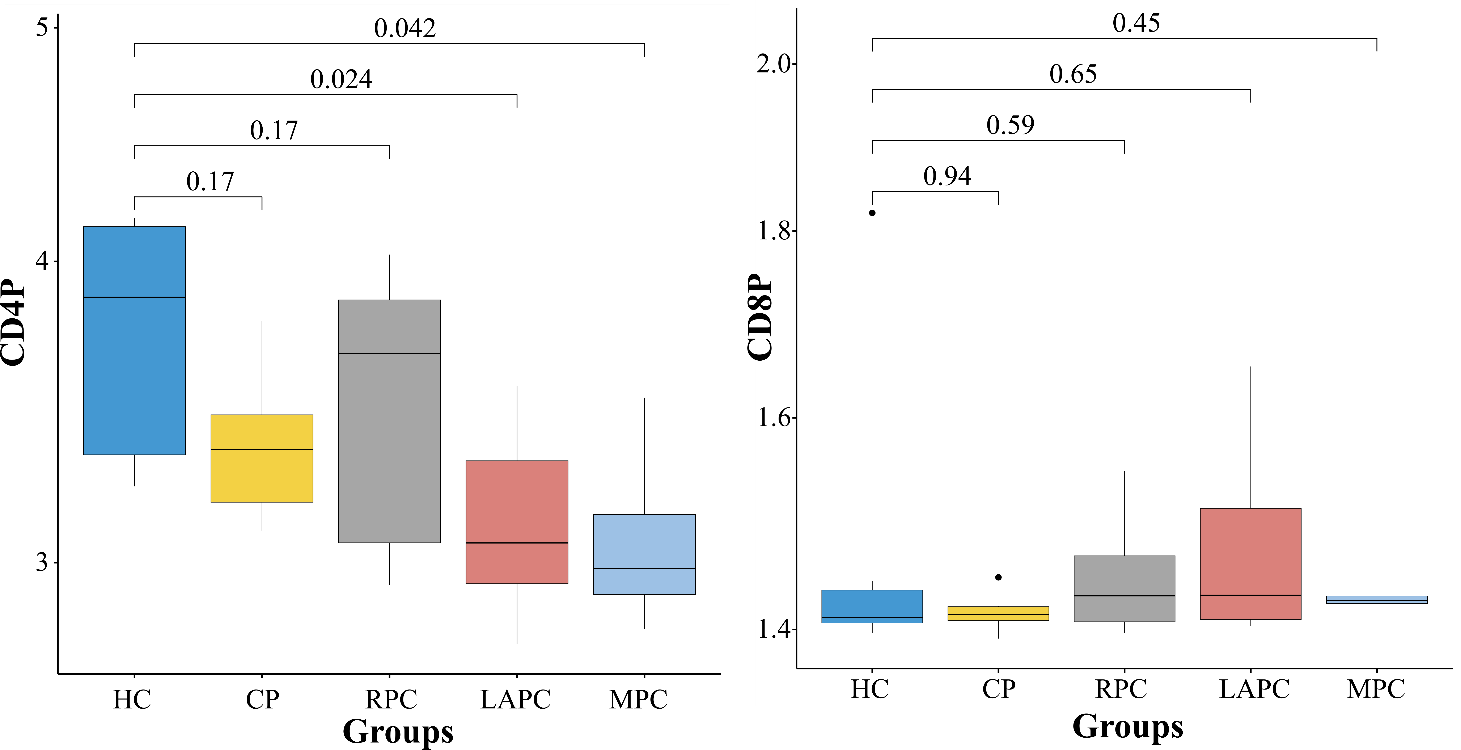


**Figure S4: Boxplots comparing the immune cell markers in PDAC plasma samples.** Enzyme**-**Linked Immunosorbent Assays (ELISA) technique was implored on the plasma samples. There were statistically significant differences observed between the PDAC groups LAPC and MPC with the control HC.


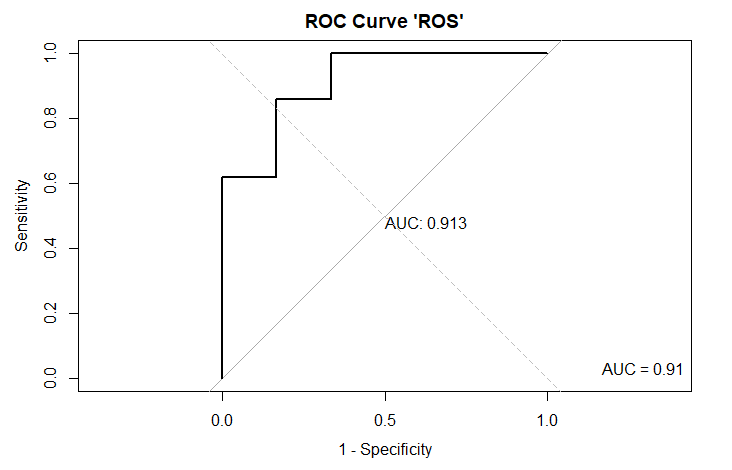


Figure S5: ROC analysis of the ROS plot. AUC of the plot was 0.91 which indicates an outstanding plot. Hence this confirms that ROS is a good marker of inflammation
