# Supplementary material for: Comparative immune profiling of pancreatic ductal adenocarcinoma progression among South African patients
Source: BMC Cancer. 2024 Jul 7;24:809. doi: 10.1186/s12885-024-12595-x (PMC11229237; doi:10.1186/s12885-024-12595-x)
Supplement: Supplementary file 2 — Supplementary Material 2 [file 12885_2024_12595_MOESM2_ESM.docx]

**SUPPLEMENTARY TABLES**

**Table S1: List of Samples and Assays**

| **Samples** | **Pathology** | **NMR** | **ROS** | **PCR** | **Immunophenotyping** | **Elisa** |
| --- | --- | --- | --- | --- | --- | --- |
| HC1 | Healthy Control |  | X | X | X | X |
| HC2 | Healthy Control | X | X | X | X | X |
| HC3 | Healthy Control | X | X | X | X | X |
| HC4 | Healthy Control | X | X | X | X | X |
| HC5 | Healthy Control | X | X | X | X | X |
| HC6 | Healthy Control | X | X | X |  | X |
| CP1 | Chronic Pancreatitis | X | X | X | X | X |
| CP2 | Chronic Pancreatitis | X | X | X | X | X |
| CP3 | Chronic Pancreatitis | X | X | X | X | X |
| CP4 | Chronic Pancreatitis | X | X | X | X | X |
| CP5 | Chronic Pancreatitis | X | X | X | X | X |
| CP6 | Chronic Pancreatitis | X | X | X |  | X |
| RPC1 | Resectable PDAC | X | X | X | X | X |
| RPC2 | Resectable PDAC | X | X | X | X | X |
| RPC3 | Resectable PDAC | X | X | X | X | X |
| RPC4 | Resectable PDAC | X | X | X | X | X |
| RPC5 | Resectable PDAC | X | X | X | X | X |
| RPC6 | Resectable PDAC | X | X | X | X | X |
| RPC7 | Resectable PDAC | X | X | X | X | X |
| RPC8 | Resectable PDAC | X | X | X | X | X |
| RPC9 | Resectable PDAC | X | X | X | X | X |
| RPC10 | Resectable PDAC | X |  | X | X | X |
| RPC11 | Resectable PDAC | X |  | X |  |  |
| RPC12 | Resectable PDAC | X |  | X |  |  |
| RPC13 | Resectable PDAC | X |  | X |  |  |
| RPC14 | Resectable PDAC | X |  | X |  |  |
| RPC15 | Resectable PDAC | X |  | X |  |  |
| RPC16 | Resectable PDAC | X |  | X |  |  |
| RPC17 | Resectable PDAC | X |  | X |  |  |
| RPC18 | Resectable PDAC | X |  | X |  |  |
| RPC19 | Resectable PDAC | X |  | X |  |  |
| RPC20 | Resectable PDAC | X |  | X |  |  |
| RPC21 | Resectable PDAC | X |  | X |  |  |
| RPC22 | Resectable PDAC | X |  | X |  |  |
| LAPC1 | Locally Advanced PDAC | X | X | X | X | X |
| LAPC2 | Locally Advanced PDAC | X | X | X | X | X |
| LAPC3 | Locally Advanced PDAC | X | X | X | X | X |
| LAPC4 | Locally Advanced PDAC | X | X | X | X | X |
| LAPC5 | Locally Advanced PDAC | X | X | X | X | X |
| LAPC6 | Locally Advanced PDAC | X | X | X | X | X |
| LAPC7 | Locally Advanced PDAC | X | X | X |  | X |
| LAPC8 | Locally Advanced PDAC | X | X | X |  | X |
| MPC1 | Metastatic PDAC | X | X | X | X | X |
| MPC2 | Metastatic PDAC | X | X | X | X | X |
| MPC3 | Metastatic PDAC | X | X | X | X | X |
| MPC4 | Metastatic PDAC | X | X | X | X | X |
|  |  |  |  |  |  |  |
|  | **Total Number of Samples** | **45** | **33** | **46** | **30** | **34** |

**Table S2****: 6-colour Flow Cytometry Immunophenotyping Panel**

| **Laser Name** | **Filter** | **Parameter** | **Emmission Wavelength** | **Lymphocytes** | **T Cells** | **NK Cells** | **Granulocytes** | **Vol per test (µl)** | **Optimised Volume (µl)** | **Catalogue #/Clone (2019)** |
| --- | --- | --- | --- | --- | --- | --- | --- | --- | --- | --- |
|  | 730/45 BP | Alexa Fluor 700 | 720 |  | CD4 |  |  | 5 | 1.25 | 561030, Clone; RPA-T4 |
| A | 780/60 BP | PE-Cy7 | 780 |  |  | CD56 |  | 5 | 1.5 | 560916; Clone; B159 |
| C | 660/20 | PE-Cy5 | 670 |  |  |  | CD16 | 5 | 1.25 | 561725, Clone; 3 G8 |
| D | 530/30BP | BB515 | 515 |  | CD57 |  |  | 5 | 1 | 565285, Clone; NK 1 |
| Power 50 | 605/12 | BV605 | 605 |  | CD8 |  |  | 5 | 1 | 564115, Clone; SK1 |
|  | 530/30 | BUV496 | 496 | CD3 |  |  |  | 5 | 1 | 612941, Clone; UCHT1 |

| **Feature** | **HC Median [IQR}** | **CP Median [IQR]** | **RPC Median [IQR]** | **LAPC Median [IQR]** | **MPC Median [IQR]** | **p-value** | **FDR** | **sig** |
| --- | --- | --- | --- | --- | --- | --- | --- | --- |
| Granulocytes | 46.2 [45.9 49.1] | 5.55 [2.29 38.4] | 67.5 [60.725 78.5] | 61.05 [53.275 61.85] | 77.65 [74.675 78.45] | 0.004 | 0.014 | ** |
| Neutrophils | 98.7 [98.4 98.8] | 63.5 [11.7 90.9] | 97.5 [96.925 97.775] | 91.6 [90.65 96.525] | 94.45 [93.675 94.55] | <0.001 | 0.007 | *** |
| Lymphocytes | 31.1 [26.6 37.8] | 7.01 [3.85 16.5] | 13.7 [10.55 17.825] | 17.55 [16.725 20.625] | 4.48 [3.865 7.14] | 0.003 | 0.014 | ** |
| T-cells | 65.2 [56.6 68.4] | 71.6 [54.8 76.3] | 61 [58.5 64.15] | 74.75 [74.35 75.225] | 51.5 [49.65 53.5] | 0.018 | 0.032 | * |
| T helper cells | 63.1 [55.8 65.7] | 58.3 [13.9 67.3] | 53 [47.225 63.15] | 49.95 [21.175 50.9] | 60.15 [53.1 67.45] | 0.127 | 0.170 |  |
| T cytotoxic cells | 41.9 [38.7 44.2] | 40.6 [29 81.4] | 36.6 [30.6 39.6] | 47.35 [46.55 75.225] | 34.55 [29.875 42.35] | 0.139 | 0.171 |  |
| T cytotoxic CD57+ | 28.8 [16.3 57.7] | 46.9 [29 47.7] | 19.75 [15.6 34.125] | 15.95 [15 19.225] | 12.65 [10.102 21.6] | 0.235 | 0.251 |  |
| Double Negative T-cells | 9.28 [7.84 9.79] | 7.42 [7.2 7.7] | 7.22 [5.482 14.075] | 3.775 [3.28 4.375] | 3.23 [3.06 4.508] | 0.006 | 0.019 | ** |
| NKTs | 0.57 [0.57 0.79] | 0.41 [0.41 0.83] | 2.29 [2.12 3.29] | 1.115 [0.9 3.46] | 3.23 [3.128 5.453] | <0.001 | 0.007 | *** |
| NKTs CD57+ | 61.5 [50 65.9] | 78.5 [12.5 96.6] | 42.5 [24.25 52.55] | 24.45 [20.575 28.325] | 35.6 [31.525 48.825] | 0.101 | 0.147 |  |
| NKTs CD56+ | 68.8 [54.5 82.4] | 0 [0 12.3] | 0.195 [0 0.623] | 0.09 [0 0.21] | 0 [0 0.51] | 0.008 | 0.021 | ** |
| NK | 2.51 [2.37 5.98] | 18.7 [9.43 52.9] | 22.55 [19.575 26.7] | 11.85 [11.25 21.9] | 27.05 [25.825 27.6] | 0.014 | 0.028 | * |
| NKCD57+ | 9.72 [5.64 21.7] | 14.1 [9.06 22] | 16.3 [5.475 22.375] | 1.1 [0.985 41.175] | 31.3 [29.775 33.5] | 0.216 | 0.247 |  |
| NKCD56bright | 12.5 [0 13.8] | 4.16 [0 34.1] | 7.84 [6.63 15.625] | 4.755 [3.46 17.42] | 8.485 [6.36 10.1] | 0.91 | 0.910 |  |
| NKCD56dimCD16+ | 26.8 [10 30.3] | 25 [22 25.4] | 63.25 [26.075 81.125] | 8.3 [3.63 62.95] | 2.58 [1.545 4.075] | 0.014 | 0.028 | * |
| NKCD56dimCD16- | 67.7 [54.2 80.3] | 36.6 [31.8 50] | 22.85 [10.65 54.075] | 65.25 [27.65 84.775] | 88.05 [86.575 89.6] | 0.024 | 0.039 | * |

**Table S3: Comparison of the total percentage of immune cells between the PDAC groups and control groups HC and CP.**
